# Genomic analyses of diverse wild and cultivated accessions provide insights into the evolutionary history of jujube

**DOI:** 10.1111/pbi.13480

**Published:** 2020-09-30

**Authors:** Mingxin Guo, Zhongren Zhang, Shipeng Li, Qun Lian, Pengcheng Fu, Yali He, Jinxin Qiao, Keke Xu, Linpei Liu, Miaoyan Wu, Zheran Du, Sunan Li, Junjie Wang, Peiyin Shao, Qiang Yu, Gan Xu, Dengke Li, Yongkang Wang, Shan Tian, Jing Zhao, Xue Feng, Ruiqiang Li, Wenkai Jiang, Xusheng Zhao

**Affiliations:** ^1^ College of Life Sciences Luoyang Normal University Luoyang China; ^2^ Jujube Research Center Luoyang Normal University Luoyang China; ^3^ Novogene Bioinformatics Institute Beijing China; ^4^ Genome Analysis Laboratory of the Ministry of Agriculture Agricultural Genomics Institute at Shenzhen Chinese Academy of Agricultural Sciences Shenzhen China; ^5^ Pomology Institute Shanxi Academy of Agricultural Sciences Taigu China

**Keywords:** jujube, resequencing, evolutionary history, seed‐setting rate, fruit weight

## Abstract

The Chinese jujube (*Ziziphus jujuba* Mill.), a member of the Rhamnaceae family, is an important perennial fruit tree crop of substantial economic, ecological and nutritional value, and is also used as a traditional herbal medicine. Here, we report the resequencing of 493 jujube accessions, including 202 wild and 291 cultivated accessions at >16× depth. Our population genomic analyses revealed that the Shanxi–Shaanxi area of China was jujube's primary domestication centre and that jujube was then disseminated into East China before finally extending into South China. Divergence events analysis indicated that *Ziziphus acidojujuba* and *Ziziphus jujuba* diverged around 2.7 Mya, suggesting the interesting possibility that a long pre‐domestication period may have occurred prior to human intervention. Using the large genetic polymorphism data set, we identified a 15‐bp tandem insertion in the promoter of the jujube ortholog of the *POLLEN DEFECTIVE IN GUIDANCE 1* (*POD1*) gene, which was strongly associated with seed‐setting rate. Integrating genome‐wide association study (GWAS), transcriptome data, expression analysis and transgenic validation in tomato, we identified a *DA3*/*UBIQUITIN‐SPECIFIC PROTEASE 14* (*UBP14*) ortholog, which negatively regulate fruit weight in jujube. We also identified candidate genes, which have likely influenced the selection of fruit sweetness and crispness texture traits among fresh and dry jujubes. Our study not only illuminates the genetic basis of jujube evolution and domestication and provides a deep and rich genomic resource to facilitate both crop improvement and hypothesis‐driven basic research, but also identifies multiple agriculturally important genes for this unique perennial tree fruit species.

## Introduction

Chinese jujube (*Ziziphus jujuba* Mill.) (2*n* = 2 × = 24), a member of the genus *Ziziphus*, in the family Rhamnaceae, is one of the most economically and ecologically important fruit trees in China. It is native to China with over 7,000 years of cultivation history and has been introduced into Korea, Japan and other neighbouring countries in 100 B.C. and then dispersed to Europe and America along the Silk Road (Qu and Wang, 1993; Liu and Wang, 2009). The dried fruits and seeds of jujubes are also valued as a traditional herbal medicine in Asian countries (Liu, 2008). Recently, a series of studies revealed that fruit extracts of jujube induced apoptotic cell death in human cancer cells (Abedini *et al*., 2016; Shin *et al*., 2018; Sun *et al*., 2013), suggesting jujube may be a promising agent to prevent or treat human cancers.

Wild jujube (*Ziziphus acidojujuba* C. Y. Cheng et M. J. Liu), also referred to as sour jujube, is regarded as the ancestor of cultivated jujube (Qu and Wang, 1993; Liu, 2006; Huang *et al*., 2016); it shows similar morphological characteristics (Figure S1) and an identical phenophase to cultivated jujube (Qu and Wang, 1993; Liu, 2006). However, unlike the arbour tree habit of cultivated jujube, most wild jujubes are bushes. Based on a survey of unearthed carbonized kernels, leaf fossils and references in ancient Chinese literature, as well as the distribution of wild and cultivated jujubes, Qu and Wang (1993) surmised that jujube is native to China and originated in the middle and lower reaches of the Yellow River and proposed that the Shanxi–Shaanxi Gorge of the Yellow River was the earliest cultivation centre (Qu and Wang, 1993). Some Chinese ancient literary texts mention jujubes, including ‘*The Book of Poetry’*, which was written about 3000 years ago and states that farmers harvested jujube fruits in early autumn. Moreover, there is archaeological evidence from excavations of ancient tombs in various locations in China, which supports that widespread jujube cultivation is several thousand years old (Qu and Wang, 1993; Liu and Wang, 2009).

**Figure 1:**
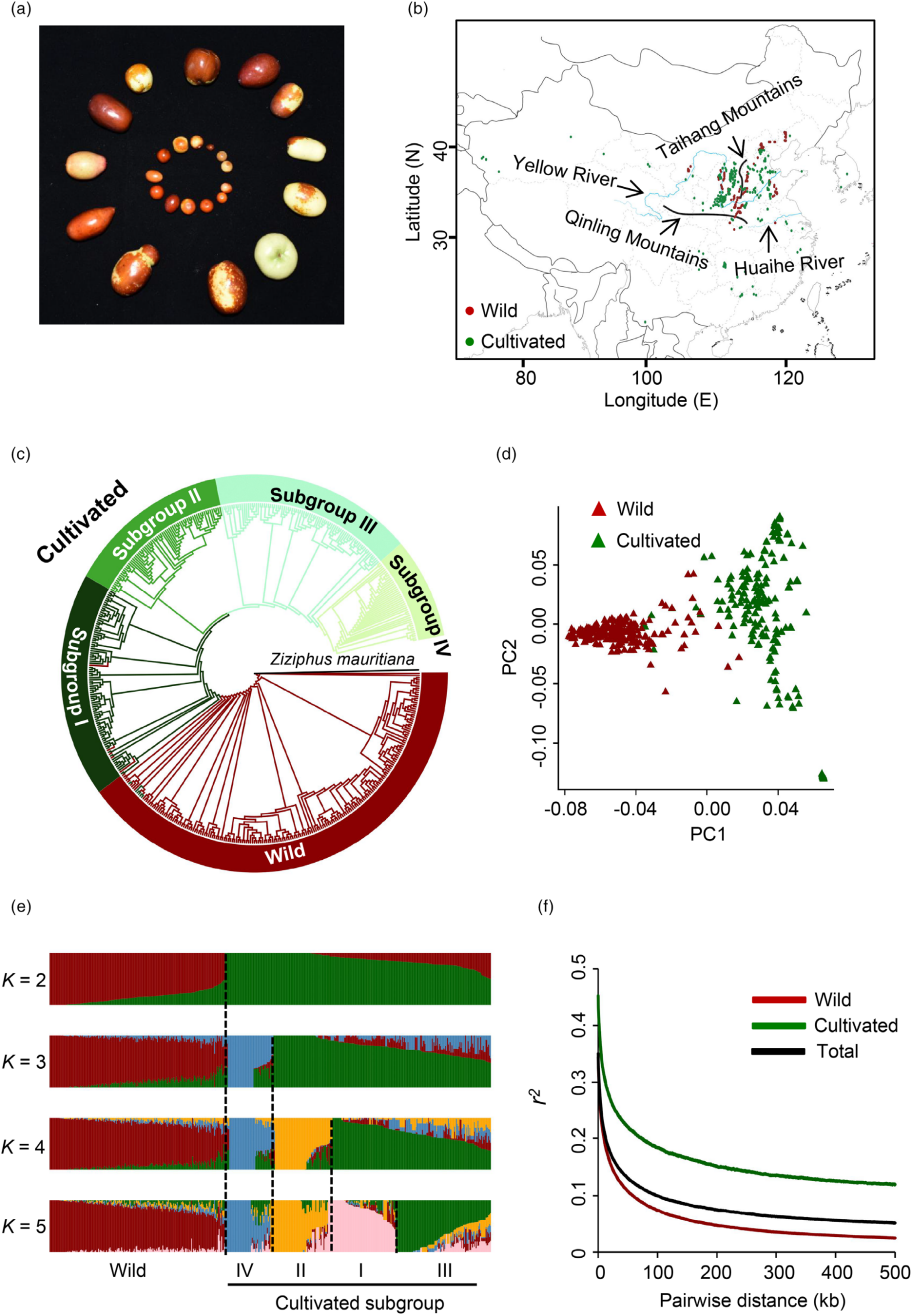
Geographic distribution and population diversity of wild and cultivated jujubes. (a) The diversity of jujube germplasms in this study. Inner circle, wild; outer circle, cultivated. (b) Geographic origins of the 493 accessions. Each dot of a given colour represents the geographic distribution of the corresponding jujube accession. (c) Phylogenetic tree of all accessions. The layered rings indicate the name of each clade. (d) Principal component analysis (PCA) plot of the first two components (PC1 and PC2) of the 493 accessions. (e) Population structure analysis of all jujube accessions with the number of ancestry kinship (*K*) ranging from 2 to 5. Each colour represents one putative ancestral background. Each vertical bar represents one accession, and the *x*‐axis shows different groups. The *y*‐axis quantifies ancestry membership. (f) Genome‐wide decay of linkage disequilibrium (LD) in wild accessions, cultivated accessions and all accessions.

Wild jujube is mainly seed‐propagated, with one or two seeds in the kernel of the fruit. However, the majority of jujube cultivars are propagated by asexual reproduction (e.g. grafting, cuttings, cultivation of root tillers and tissue culture), because many modern cultivars produce few or no seeds and/or are self‐incompatible or difficult to cross (Huang *et al*., 2016). Asexual reproduction enables breeders to fix valuable agronomic traits more rapidly in cultivated jujube. The reproductive system transition that occurred in jujube resembles that of most other perennial fruit crops, over 75% of which are clonally propagated (Miller and Gross, 2011). Selection‐driven increases in fruit or grain size are referred to as the ‘domestication syndrome’, and these phenotypic differences distinguish most fruit crops or seed‐propagated annual crops from their progenitors (Doebley *et al*., 2006). The jujube cultivars’ fruits are much bigger than wild individuals (Figure 1a). Recently, resequencing of wild and cultivated woody perennial fruit crops has allowed researchers to identify horticultural impactful genes and/or to clarify the evolutionary origins and domestication trajectories of species including citrus (Wang *et al*., 2018; Wang *et al*., 2017; Wu *et al*., 2018), apple (Duan *et al*., 2017), pear (Wu *et al*., 2018), peach (Cao *et al*., 2019; Li *et al*., 2019; Yu *et al*., 2018) and pistachio (Zeng *et al*., 2019).

In this study, we performed deep genome resequencing of 493 wild and cultivated jujube accessions and analysed their genomic variation dynamics during evolutionary process. Population genomic analyses indicated that Shanxi–Shaanxi area was the primary domestication centre, and we found evidence for a subsequent easterly and then southerly dispersal pattern for cultivated jujubes. Analysis of the divergence time between *Z*.* acidojujuba* and *Z*.* jujuba* revealed a possible long pre‐domestication period led to the morphological differentiation of wild and cultivated jujubes. Using multiple strategies, we identified a candidate gene, ortholog of *POD1*, as being significantly associated with the seed‐setting rate and an ortholog of *DA3*/*UBP14*, which is a key regulator determining fruit weight in jujube. Our large‐scale resequencing study provides a deep genomic data resource and reveals insights about the genetic basis of important fruit quality traits in cultivated jujubes and will thus facilitate future basic research and jujube improvement breeding programmes.

## Results

### Genome resequencing and variation calling

A total of 493 jujube accessions, including 202 wild jujube individuals (*Z*.* acidojujuba*) and 291 jujube cultivars (*Z*.* jujuba*), were examined in this study (Figure 1b and Table S1). Among the 493 accessions, 292 were resequenced as part of our recent study focusing on dissecting the genetic basis of seven domestication traits in jujube (Guo *et al*., 2020). Resequencing of the 493 jujube accessions by Illumina HiSeq PE150 generated a total of 3.11 trillion bp of sequence, with an average depth of 16× and coverage of 96.24% of previously reported jujube genome assembly (Table S2) (Huang *et al*., 2016). After mapping against the cultivated jujube ‘Junzao’ reference genome (Huang *et al*., 2016), we identified 12 414 260 single nucleotide polymorphisms (SNPs) and 1 203 889 indels (shorter than or equal to 5 bp) (Table S3). Among the identified SNPs, 2 244 346 (18.08%) were located in genes, and the frequency of such genetic variants was more than twofold higher in noncoding regions (70.19%; 1 575 261) than in coding regions (29.81%; 669 085) (Table S3).

Within coding regions, nonsynonymous SNPs (391 634) were more frequent than synonymous SNPs (264 958); this 1.48 ratio of nonsynonymous‐to‐synonymous substitutions in jujube is higher than that in *Arabidopsis thaliana* (0.83) (Clark *et al*., 2007), pigeon pea (1.18) (Varshney *et al*., 2017), rice (1.29) (Xu *et al*., 2011), and peach (1.31) (Cao *et al*., 2014). After filtering (coverage depth ≥ 6, MAF ≥ 0.05 and miss rate ≤ 0.1), a total of 1 952 561 high‐quality SNPs with an average density of 5.56 SNPs per kilobase were obtained for subsequent analysis. PCR amplification and Sanger sequencing on genomic regions containing 400 randomly selected SNPs indicated a high accuracy rate (99.25%) for our SNP calling (Table S4).

### Jujube population structure and genetic diversity

We explored the phylogenetic relationships among the 493 accessions based on our 1 952 561 high‐quality SNPs, using Indian jujube as the outgroup (we sequenced one *Ziziphus mauritiana* individual to facilitate this analysis). The resulting neighbour‐joining tree strongly supported the distinct clustering of the 493 accession into our two specified wild vs. cultivated groups. Moreover, consistent with previous reports (Qu and Wang, 1993; Liu, 2006; Huang *et al*., 2016), the tree emphasized again that cultivated jujubes were domesticated from wild jujubes (Figure 1c). A principal component analysis (PCA) based on the SNP data illustrated a similar pattern of two distinct clusters for the wild (*Z*.* acidojujuba*) and cultivated (*Z*.* jujuba*) accessions (Figure 1d).

**Figure 2:**
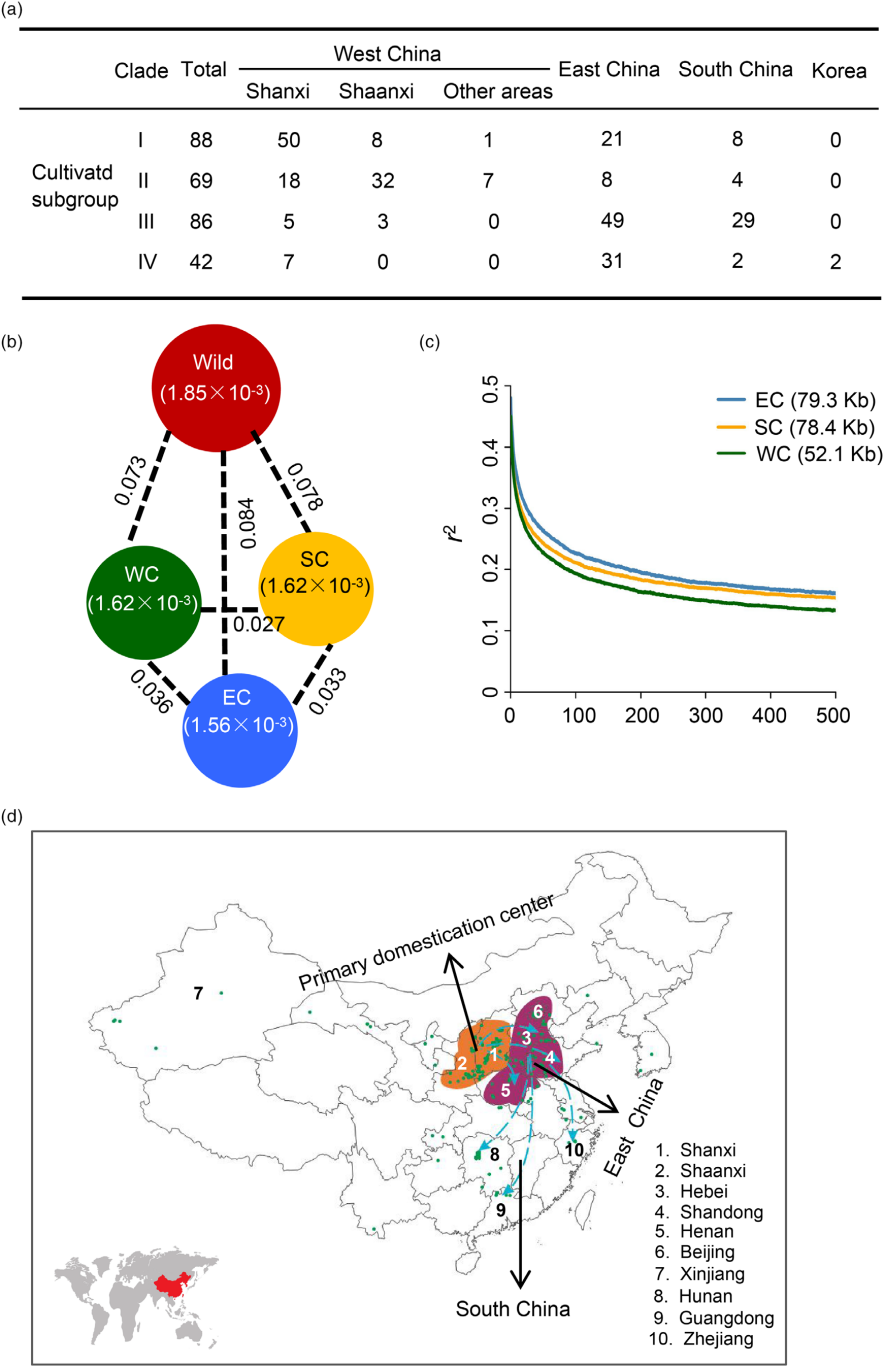
The domestication centre and dispersal routes of Chinese jujube. (a) Geographic origins of cultivated subgroups I, II, III and IV. (b) Nucleotide diversity (*π*) of wild group and cultivated jujubes native to West, East and South China and population differentiations (*F*_ST_) between two groups. The value in each circle represents the nucleotide diversity of this group, and the value on each line shows the population differentiation between the two groups. (c) Genome‐wide decay of linkage disequilibrium (LD) in cultivated jujubes native to West, East and South China. The extents of LD decay of three cultivated subgroups were indicated in brackets. (d) The primary domestication centre and dispersal routes of jujubes.

The phylogenetic analysis also supported divergence of the cultivated accessions into four subgroups that reflected plausible geographic distribution patterns (Figure 2a and Table S1). Cultivated subgroup I mainly contained accessions from West China (on the west side of the Taihang Mountains) and were biased towards Shanxi Province. Cultivated subgroup II mainly consisted of the accessions also from West China, with enrichment of accessions from Shaanxi Province. Cultivated subgroup III mainly included the accessions from East China (on the east side of the Taihang Mountains) and South China (on the south side of the Qinling Mountains–Huaihe River Line, which is the watershed between the South and North China). Cultivated subgroup IV was biased towards accessions from East China and South Korea (Figure 2a).

To further assess the genetic relationships among these accessions, we performed a structure analysis using ADMIXTURE with *K* values ranging from 2 to 5 (Figure 1e). At *K* = 2, the accessions were clearly divided into the same wild and cultivated groups identified in the phylogeny and PCAs. At *K* = 3, separation of many accessions from the aforementioned cultivated subgroup IV became apparent, while at *K* = 4, further separation of the cultivated subgroup II accessions was evident. By *K* = 5, the remaining cultivated accessions were separated to groups that closely matched subgroups I and III.

The nucleotide diversity (*π*) levels of the wild and cultivated groups were 1.85 × 10^−3^ and 1.62 × 10^−3^, respectively. In comparison with other perennial crops, the nucleotide diversity of jujubes is higher than peach (1.0 × 10^−3^; Li *et al*., 2019), but lower than that of date palm (9.2 × 10^−3^) (Hazzouri *et al*., 2015), apple (~2.20 × 10^−3^) (Duan *et al*., 2017) and pear (5.5 × 10^−3^) (Wu *et al*., 2018). The similar levels of nucleotide diversity that we detected for the jujube cultivar group and the progenitor wild jujubes indicated that the jujube domestication process apparently only involved a very weak bottleneck, findings consistent with reports for other perennial fruit crops including apple and pear (Duan *et al*., 2017; Wu *et al*., 2018). Further indicating relatively low genetic differentiation between wild progenitor and cultivated jujubes, the fixation index value (*F*_ST_) between the resequenced wild and cultivated groups was only 0.084; for comparison, the *F*_ST_ was ~0.28 between wild and cultivated citrus populations (Wang *et al*., 2017) and ~0.76 between wild and domesticated peaches (Li *et al*., 2019).

Decay of linkage disequilibrium (LD; indicated by *r* ^2^) is typically measured as the genomic distance when LD has decreased to half of its maximum value. We found that the extent of LD decay in the wild, cultivated and total jujube accessions was 15.7 kb (*r* ^2^ = 0.17), 45.2 kb (*r* ^2^ = 0.23) and 20.5 kb (*r* ^2^ = 0.18), respectively (Figure 1f), values which are substantially higher than those detected for peach and pear (Cao *et al*., 2014; Li *et al*., 2019; Wu *et al*., 2018).

### Shanxi–Shaanxi area was the primary centre of jujube domestication

Our phylogenetic analysis indicated that cultivated subgroup I is the closest to the wild group, followed by subgroup II (Figure 1c). Most of the accessions for subgroups I and II are from West China, with a clear bias towards Shanxi and Shaanxi provinces, while the remaining small proportion of the jujube cultivars are from East China and South China (Figure 2a) and comprise cultivated subgroups III and IV, both of which are apparently relatively far from the wild group accessions in our phylogenetic tree (Figure 1c).

We evaluated the *π* values and LD decay of accessions from West China, East China and South China, which showed that the nucleotide diversity of West China and South China is slightly higher than that of East China (Figure 2b), and revealed that the West China accessions exhibit more rapid LD decay than accessions from East and South China (Figure 2c). We further computed the *F*_ST_ values between wild accessions and three cultivated subgroups from West China, East China and South China, which showed that the *F*_ST_ value between wild and West China accessions was the lowest (Figure 2b). These results also support that the accessions from West China are more closely related to the wild accessions than accessions from other areas. These results led us to conclude that jujube was originally domesticated in West China (Figure 2d).

Supporting this conclusion, the Shanxi and Shaanxi provinces in West China both have extensive wild and cultivated germplasm resources (Figures 1b and 2d; Table S1), and it is notable that there are still old wild and cultivated jujubes (about 1,000 years old) grown in Shanxi and Shaanxi provinces (Qu and Wang, 1993; Liu and Wang, 2009). Further, given that most accessions native to Shanxi were classified in cultivated subgroup I while most accessions native to Shaanxi were classified into cultivated subgroup II, our data support the idea that domestication may have commenced in Shanxi earlier than in Shaanxi.

Our data also largely support the dissemination of domesticated jujube from the primary domestication centre in the Shanxi–Shaanxi area into East China (Figure 2d), and both our phylogenetic tree and population structure analyses indicate that most of the accessions from South China are grouped alongside those from East China (Figures 1e and 2a), suggesting further dissemination to the South via East China (Figure 2d).

### Divergence events and demographic history

**Figure 3:**
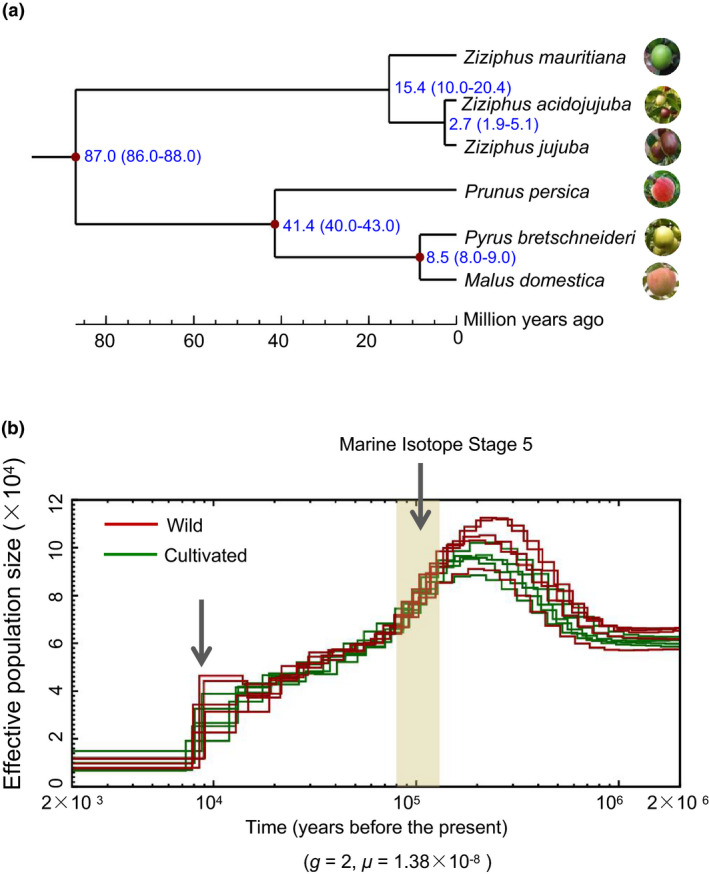
Divergence times and demographic history of jujube species. (a) Estimation of divergence times between *Ziziphus jujuba* and other five plant species. The blue numbers on the nodes are the divergence times, and numbers in brackets are the 95% confidence intervals. (b) Demographic histories of the wild and cultivated jujubes reconstructed using the PSMC model for the effective population size (*Ne*). *g* (generation time) = 2 years and *μ* (neutral mutation rate per generation) = 1.38 × 10^−8^.

We next explored the evolutionary history of jujube by predicting the divergence times of six species (Figure 3a), including the aforementioned three Ziziphus species from the Rhamnaceae family and three Rosaceae species (*Prunus persica*, *Pyrus bretschneideri* and *Malus domestica*). An MCMCtree analysis estimated these six species diverged from a common ancestor about 87.0 Mya (95% CI: 86.0–88.0) (Figure 3a). Subsequently, *Z*.* mauritiana* and *Z*.* acidojujuba* diverged about 15.4 Mya (95% CI: 10.0–20.4) (Figure 3a). Fascinatingly, this predicted divergence date is supported by an archaeological discovery from Linju in Shandong Province of East China that a *Z*.* miojujuba* leaf fossil (12–14 Mya) exhibits very similar leaf morphology to modern wild jujube (Qu and Wang, 1993, Liu and Wang, 2009).

The MCMCtree also estimated that *Z*.* acidojujuba* and *Z*.* jujuba* diverged about 2.7 Mya (95% CI: 1.9–5.1) (Figure 3a). Intriguingly, the Taihang Mountains, which divided North China into West China and East China, were uplifted and eventually formed at a similar period (early Quaternary Period, ~2.6 Mya; Wu *et al*., 1999). The altitudes in West China are dramatically higher than East China, which topologically is a large plain at low altitude. Our data thus support that the Shanxi–Shaanxi area in West China was very likely the primary domestication centre of jujube. We cannot decisively conclude that the uplift of the Taihang Mountains led to the speciation of ancient jujubes in the area to the west of these mountains, but this scenario seems both geologically and genomically plausible. The divergence time of *Z*.* acidojujuba* and *Z*.* jujuba* occurred far prior to any possible human intervention, indicating that the morphological divergence of these species was apparently mediated by natural factors such as natural mutations and natural hybridizations. Similar long periods in the evolutionary histories of other crops have been referred to as 'pre‐domestication' phases (Yu *et al*., 2018), and extensive evidence implicates that contributions from animals including primates have shaped the evolutionary history of fruit species in such phases (Yu *et al*., 2018).

To explore the demographic history of jujube, we firstly estimated the effective population size (*N*_e_) (2 × 10^3^–2 × 10^6^ years ago) using a pairwise sequentially Markovian coalescent (PSMC) method (Li and Durbin, 2011). The wild and cultivated groups exhibited similar demographic histories (Figure 3b): both groups reached the maximum *N*_e_ at ~200 Kya (thousand years ago), while the *N*_e_ decreased continuously after 200 Kya. And the *N_e_* of two groups experienced two dramatic decline periods. One period occurred during Marine Isotope Stage 5 (80–130 Kya), which corresponds to the last major interglacial period (Lisiecki and Raymo, 2005; Jouzel *et al*., 2007), while the other period occurred about 8–9 Kya (Figure 3b). These two sharp reductions likely resulted from the climate changes, which occurred at the end of the last ice age (Dong *et al*., 2017). Our observation that the *N_e_* of wild and cultivated groups was basically identical throughout most of the examined demographic trajectories further supports our aforementioned speculation about relatively low overall genetic diversity and about a very weak bottleneck during the domestication of jujube.

### Genome‐wide selective sweep signals

**Figure 4:**
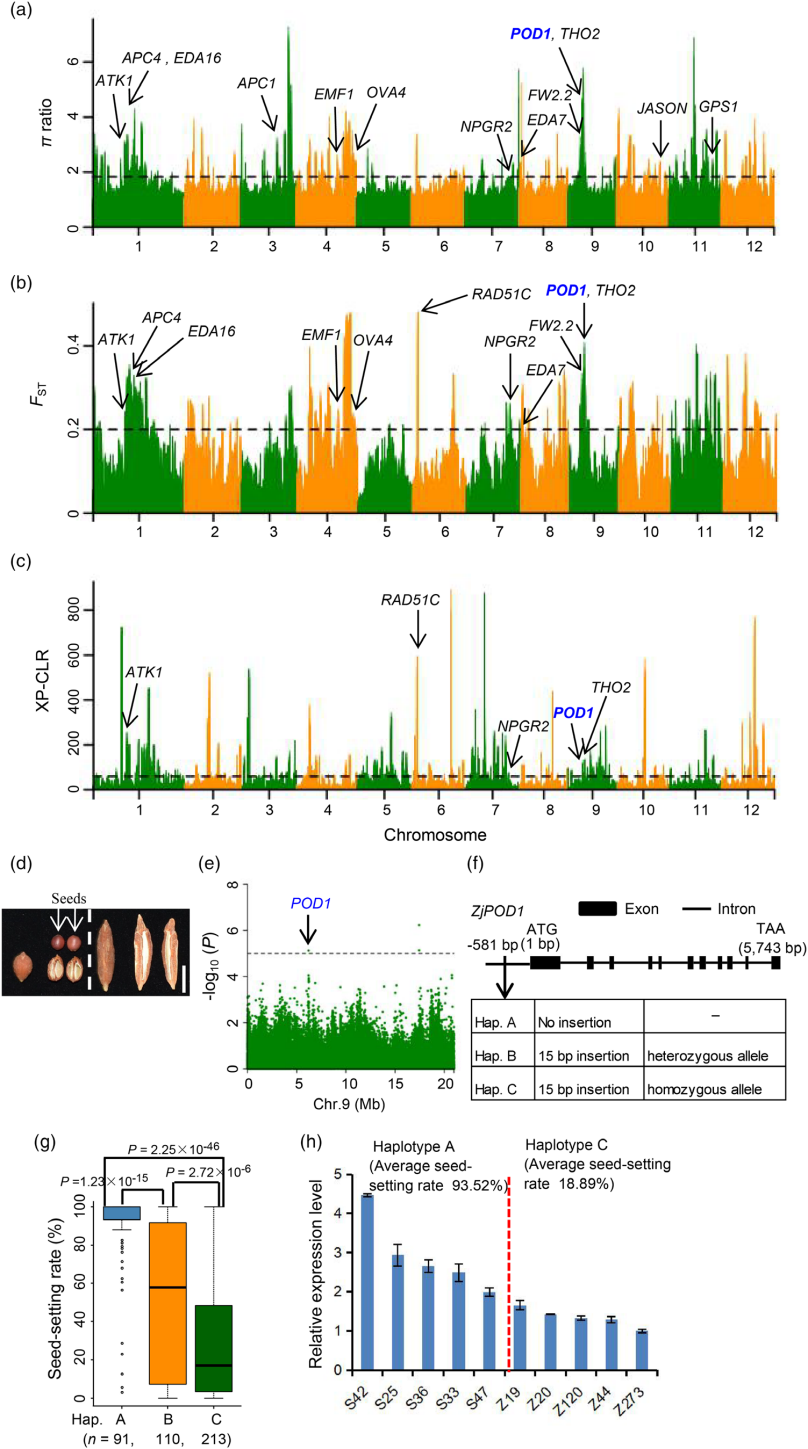
Genome‐wide distributions of selective sweeps in jujube and identification of candidate genes associated with seed‐setting rate. (a) Selective sweeps were identified by *π*_w/_ *π*_c_. The horizontal black line corresponds to the top 5% (≥1.83) of the *π*_w/_ *π*_c_ values. (b) Highly divergent regions between wild and cultivated jujubes. The horizontal black line corresponds to the top 5% (≥0.20) of the differentiation signals. (c) Genome‐wide distributions of XP‐CLR values. The horizontal black line corresponds to the top 5% (≥59.80) of the XP‐CLR values. In (a), (b) and (c), some candidate genes potentially regulating reproductive system development in selection regions are shown. (d) Phenotypes of intact kernels and longitudinal sections of wild (left) and cultivated (right) jujubes. Bar = 1 cm. (e) Manhattan plot of the seed‐setting rate trait on the chromosome 9 of jujube.(f) Gene structure and DNA polymorphisms in *ZjPOD1*. (g) Box plots for seed‐setting rate based on the three haplotypes of *ZjPOD1*. Centre bold lines indicate the median, and box limits represent the upper and lower quartiles. Whiskers extend to data no more than 1.5 times the interquartile range, and dots represent outliers. *n* indicates the number of accessions with the same haplotype. Significant differences were determined by two‐tailed Welch’s *t*‐test. (h) Relative expression patterns of the *ZjPOD1* in young fruits (~5 mm) of two types of accessions (haplotypes A and C). Values represent mean ± SEM of triplicates for qPCR.

To identify genomic regions associated with jujube domestication, we used three approaches including the *π* ratio, *F*_ST_, and XP‐CLR (the cross‐population composite likelihood ratio) (Chen *et al*., 2010) to compare the wild and cultivated groups. The top 5% of the genomic windows or regions with high values in each comparison were defined as selective sweeps. Finally, there were 263, 197 and 271 selective sweeps identified based on the *π* ratio, *F*_ST_, and XP‐CLR, respectively (Figure 4a–c and Table S5). These selective sweeps covered 27.27 Mb (7.77%), 24.30 Mb (6.92%) and 27.81 Mb (7.92%) of the assembled genome, harbouring 2192, 1692 and 1998 genes, respectively (Table S6). Confirming the selection signals and narrowing down our list of candidate genes, we found that 993 candidate genes were common to both the *π*_w/_ *π*_c_ (w, wild; c, cultivated) and *F*_ST_ results (Table S6).

Among these 993 genes, a protein kinase cluster including 5 genes was located in the strongest signal of a selective sweep on Chr.3 identified by *π* ratio analysis (Table S6). Intriguingly, an *EARLY FLOWERING 3* ortholog (*Zj*.*jz000799141*) encoding the flowering time control protein was probably under selection (Table S6). In *Arabidopsis*, EARLY FLOWERING 3 (AT2G25930) plays a critical role in the regulation of flowering time (Hicks *et al*., 2001). This is consistent with the observation that most of the wild jujubes in this study showed earlier flowering than the cultivars. We used the Gene Ontology (GO) tools to functionally annotate these 993 candidate genes, which revealed that for the ‘biological process’ category, six out of the eight most highly enriched categories (*P* < 0.05) were associated with plant reproduction processes (Figure S2 and Table S7), including ‘multi‐organism reproductive process’, ‘pollination’, ‘pollen–pistil interaction’, ‘recognition of pollen’, ‘reproductive process’ and ‘reproduction’.

### A striking change in reproductive strategy during jujube domestication

As with several other perennial fruits such as citrus and banana, the sexual‐to‐asexual shift in reproductive modes is a striking feature of jujube (Qu and Wang, 1993) (Figures 4d and Figure S3). A batch of candidate genes potentially associated with reproductive development were identified through selective sweep analyses (Figure 4a‐c and Table S8). These candidates included *Zj*.*jz015743041* (*POD1* ortholog) (Li *et al*., 2011), *Zj*.*jz015743038* (*THO2* ortholog) (Francisco‐Mangilet *et al*., 2015), *Zj*.*jz007373109* (*NPGR2* ortholog) (Golovkin et al., 2003), *Zj*.*jz037653027* (*RAD51C* ortholog) (Abe *et al*., 2005) and *Zj*.*jz017713035* (*APC4* ortholog) (Wang *et al*., 2012). The orthologs of these genes in *Arabidopsis* have been demonstrated to regulate female gametogenesis, pollen germination, pollen tube growth and embryogenesis.

We performed GWAS for seed‐setting rate (kernels with full seeds/all detected kernels) and found that one SNP located within the intron of *POD1* ortholog (*ZjPOD1*, *Zj*.*jz015743041*) was strongly associated with seed‐setting rate (Figures 4e and Figure S4). Moreover, *ZjPOD1* was simultaneously identified by all three selective sweep detection approaches, which showed very high *π*_W/_ *π*_C_ and *F*_ST_ values (Figure 4a,b and Table S8). In *Arabidopsis*, POD1 (AT1G67960) regulates pollen tube guidance and early embryo patterning (Li *et al*., 2011). Thus, we focused on *ZjPOD1* and looked at other genetic variations including indel. Finally, we found that a 15‐bp tandem insertion in the *ZjPOD1* promoter region was highly associated with seed‐setting rate (Figure 4f,g). PCR amplification and Sanger sequencing were conducted to validate this variation, and these results were consistent with our resequencing data (Figure S5). This 15‐bp tandem insertion formed three haplotypes (A–C) (Figure 4f): the accessions carrying haplotype A exhibited significantly higher seed‐setting rates than did those with haplotype B, and the haplotype B accessions in turn had significantly higher seed‐setting rates than haplotype C accessions (Figure 4g). We used two types of accessions (haplotypes A and C) to perform *ZjPOD1* expression analysis in young fruits (~5 mm). The gene displayed higher expression in accessions carrying haplotype A than in accessions carrying haplotype C (*P* = 0.008) (Figure 4h). These results indicated that *ZjPOD1* is an important candidate gene regulating seed‐setting rate in jujube.

### Fruit enlargement prior to and during jujube domestication

**Figure 5:**
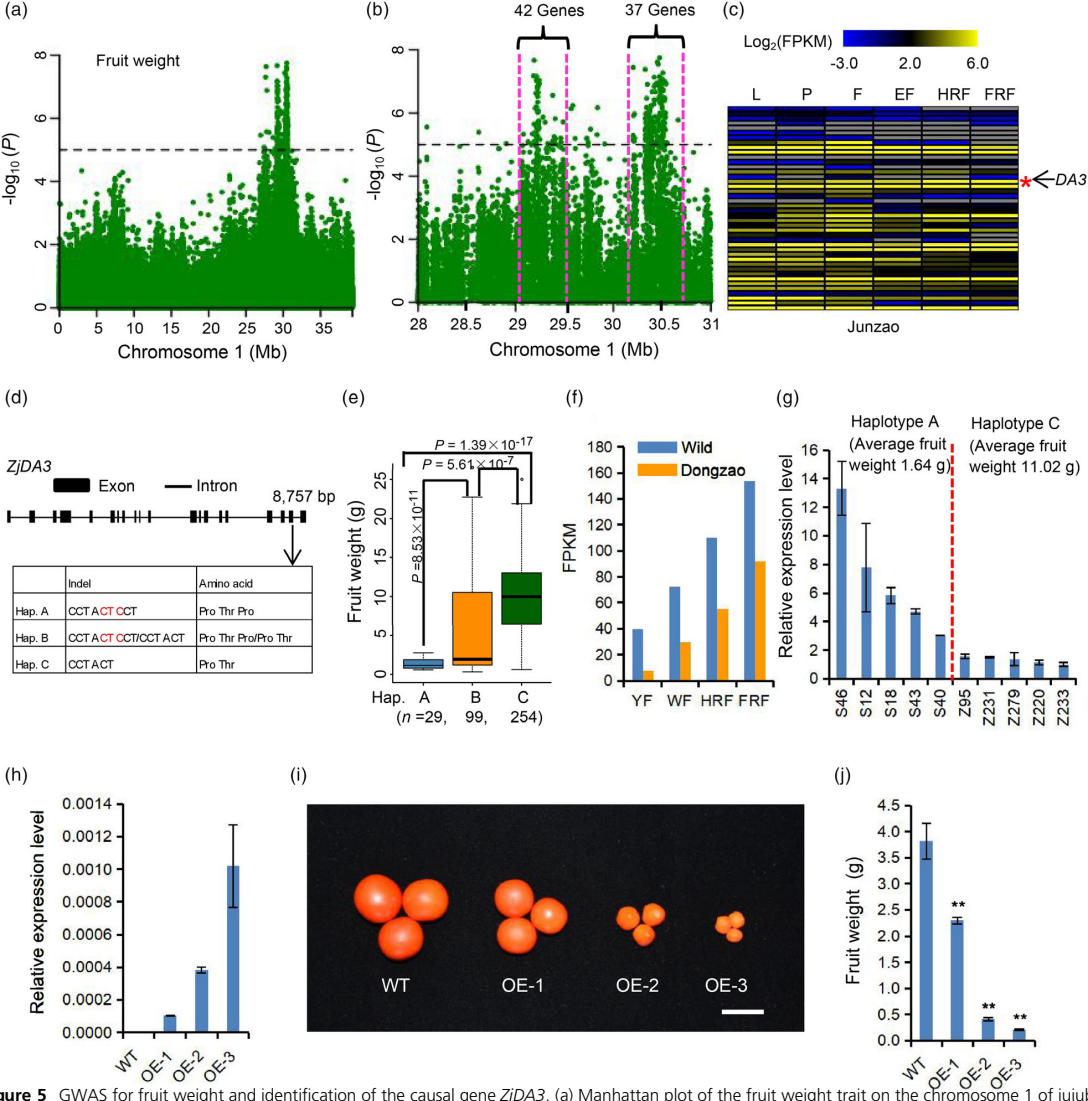
GWAS for fruit weight and identification of the causal gene *ZjDA3*. (a) Manhattan plot of the fruit weight trait on the chromosome 1 of jujube. (b) Two adjacent GWAS peaks were identified harbouring 42 and 37 genes, respectively. (c) Comparative tissue‐specific transcript profiles of 42 genes in the first GWAS peak in cultivar ‘Junzao’. Colour scale represents FPKM‐normalized log_2_‐transformed counts. (d) Gene structure and DNA polymorphisms in *ZjDA3*. (e) Box plots for fruit weight based on the three haplotypes of *ZjDA3*. Centre bold lines indicate the median, and box limits represent the upper and lower quartiles. Whiskers extend to data no more than 1.5 times the interquartile range, and dots represent outliers. *n* indicates the number of accessions with the same haplotype. Significant differences were determined by two‐tailed Welch’s *t*‐test. (f) RNA‐seq data reveal the transcript levels of *ZjDA3* in both one wild accession and one cultivar ‘Dongzao’ at four fruit developmental stages. (g) Relative expression patterns of *ZjDA3* in enlargement fruits of two types of accessions (haplotypes A and C). Values represent mean ± SEM of triplicates for qPCR. (h) Transcript levels of *ZjDA3* in the wild‐type (WT) tomato and three overexpression transgenic lines, detected by qPCR. Data shown are mean values of three technical repeats with SEM. (i) Tomato fruit morphology of wild‐type and three overexpression of *ZjDA3* lines, Bar = 2 cm. (j) Statistical data of tomato fruit weight in wild‐type and three overexpression of *ZjDA3* lines. Values are means ± SEM., *n* = 8. ‘**’ indicated *P* < 0.01. Abbreviations: L, leaf; P, phloem; F, flower; YF, young fruit; EF, enlargement fruit; WMF, white mature fruit; HRF, half‐red fruit; FRF, full‐red fruit.

Fruit enlargement has apparently a dominant aspect of jujube domestication (Figures 1a and Figure S3); however, little is known about the genetic basis underlying fruit enlargement. Our selective sweep analyses identified a *FW2*.*2*/*CNR1* ortholog (*Zj*.*jz029849045*) and 12 cyclin family genes, which may help regulate jujube fruit size (Table S8). The *FW2*.*2* gene in tomato (Frary *et al*., 2000) and its closest homolog in maize, *CNR1* (Guo *et al*., 2010), have been reported to control fruit and ear size, respectively. Seeking additional candidate genes associated with fruit weight, we performed GWAS using the resequencing data for 382 accessions (no phenotypic data for the other 111 accessions). Two adjacent GWAS peaks were identified on chromosome 1 (Figures 5a,b and S6a). One GWAS peak spanned from 29.03 to 29.46 Mb and harboured 42 genes (Table S9). The other peak spanned from 30.15 to 30.72 Mb and harboured 37 genes (Table S9).

We also conducted a subsequent GWAS using approximately 350 accessions for which we had phenotypic data from multiple years, which identified a single peak on chromosome 1 from 29.03 to 29.46 Mb (Figure S6). We therefore focused on this GWAS peak and analysed the transcript levels for its 42 putative genes at different fruit developmental stages in one cultivar ‘Junzao’ (Figure 5c). It was notable that an ortholog of an *Arabidopsis* ubiquitin‐specific protease (*DA3*/*UBP14*; *AT3G20630*) that is known to regulate the size of organs including the endosperm, cotyledons, leaves and flowers (Xu *et al*., 2016) was highly expressed during fruit maturation in cultivar ‘Junzao’.

Analysis of the genetic variations for the *DA3/UBP14* ortholog (*ZjDA3*; *Zj*.*jz038707057*) among our jujube accessions indicated that no nonsynonymous SNP in coding or promoter regions was significantly associated with fruit weight. We then analysed the indels (1–50 bp) in *ZjDA3* and found that a three‐bp (CTC) deletion was highly associated with fruit weight (Figure 5d,e). The results of PCR amplification and Sanger sequencing were consistent with resequencing (Figure S5). This three‐bp deletion led to an amino acid (proline) deletion in two of the three haplotype groups it defined (Figure 5d). The fruit weights of the 7.59% of accessions of the haplotype harbouring the full‐length sequence were significantly lower than for the accessions lacking proline at amino acid position 726 (Figure 5e). Furthermore, all of the *Z*. *jujuba* cultivars assessed in our GWAS harboured the deletion allele, indicating very strong directional selection for the deletion during jujube domestication.

We analysed the transcript levels of *ZjDA3* in both one wild accession and one cultivar ‘Dongzao’ at four fruit developmental stages using RNA‐seq data. The results revealed that *ZjDA3* showed higher transcript levels in wild accession than in cultivar ‘Dongzao’ (Figure 5f). We then used two types of accessions (haplotypes A and C) to perform *ZjDA3* expression analysis in enlargement fruits. The gene displayed higher expression in accessions carrying haplotype A than in accessions carrying haplotype C (Figure 5g). These results indicated that *ZjDA3* may be a negative regulator determining fruit weight, which was similar to *DA3* in *Arabidopsis*. Loss of function of *DA3* increases organ size in *Arabidopsis* (Xu *et al*., 2016).

To validate the functions of *ZjDA3*, we constructed the *35S::ZjDA3^Hap^* ^.^ *^A^* binary plant transformation vector and generated overexpression tomato lines. Three overexpression transgenic lines (OE‐1, OE‐2 and OE‐3) were selected for phenotypic analysis. The expression level of *ZjDA3* in above three OE lines was obviously up‐regulated (Figure 5h). When we detected the fruit size and weight, we found that the fruit size and weight of three OE lines have significantly decreased compared with wild‐type tomato (Figures 5i,j and Figure S7). Therefore, we conclude that *ZjDA3* negatively regulates fruit size and weight in jujube.

### Divergent selection of fruit taste among cultivated jujubes

**Figure 6:**
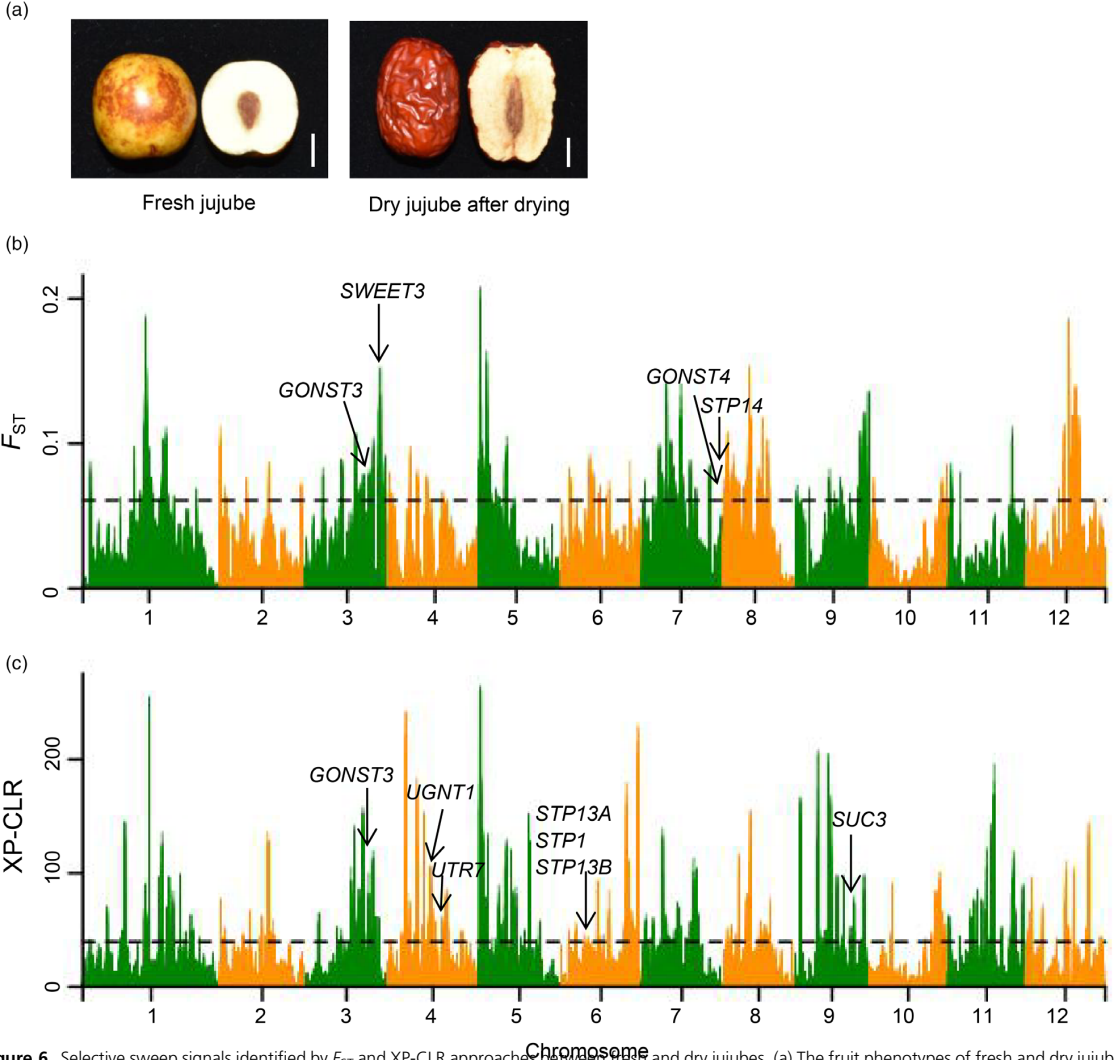
Selective sweep signals identified by *F*_ST_ and XP‐CLR approaches between fresh and dry jujubes. (a) The fruit phenotypes of fresh and dry jujubes. Bars = 1 cm. (b) Highly divergent regions between fresh and dry jujubes. The horizontal black line corresponds to the top 5% (≥0.061) of the differentiation signals. (c) Genome‐wide distributions of XP‐CLR values. The horizontal black line corresponds to the top 5% (≥39.56) of the XP‐CLR values. In (b) and (c), some candidate genes potentially regulating fruit sweetness in selection regions are shown. Gene abbreviations: *GONST,* Golgi nucleotide sugar transporter; *UGNT,* UDP‐GlcNac transporter; *UTR,* UDP‐galactose transporter; *STP,* sugar transport protein; *SUC,* sucrose transporter.

Cultivated jujubes are divided into two major categories based on how their fruits are typically consumed: fresh jujube and dry jujube. At harvest, (half‐red or full‐red fruit), fresh jujubes taste sweet and crisp, with an apple‐like flesh texture, whereas dry jujubes have a rough flesh and poor eating quality at this stage. In contrast, dry jujubes are highly desirable after they have been properly dried, when they have soft flesh inside a papery skin, similar to some forms of palm dates (Figure 6a). Seeking to dissect the genetic basis of the phenotypic divergence among these two types, representative accessions from two categories (including 43 fresh jujubes and 50 dry jujubes) were chosen (Table S10) and both *F*_ST_ and XP‐CLR analyses were used for comparison. By selecting the regions with the top 5% highest values, 201 and 273 selective sweeps were detected, respectively, for fresh and dry jujubes (Table S11).

As the genes in these regions may regulate the phenotypes that distinguish between dry and fresh jujubes (Table S12), we focused on genes with annotations, suggesting that they may influence sugar content and thusly identified 10 selective‐sweep‐bearing genes, which putatively encode sugar transporter proteins (Figure 6b,c and Table S13). Fruit crispness is another distinguishing trait between fresh and dry jujube. It has been reported in strawberry, peach and apple that pectin is a key factor in fruit crispness (Atkinson *et al*., 2012; Quesada *et al*., 2009; Yang *et al*., 2009). We found that some of the selective‐sweep‐bearing genes from our comparison of dry and fresh jujubes encode enzymes known to function in pectin metabolism in fruits including pectinesterases, polygalacturonases, pectin methylesterases, pectin lyases and galacturonosyltransferases (Table S13), potentially helping explaining the texture divergence of fresh and dry jujubes.

## Discussion

We resequence nearly 500 wild and cultivated jujube accessions and conducted a variety of genetic diversity, demography, quantitative genetics and molecular genetics analyses to uncover biological and historical insights underlying the evolutionary domestication histories of this important perennial fruit crop. Unlike annual crops such as soy bean (Zhou *et al*., 2015), cucumber (Qi *et al*., 2013), tomato (Lin *et al*., 2014) and melon (Liu *et al*., 2020), and other perennial fruit crops such as peach (Li *et al*., 2019) that showed a sharp reduction in nucleotide diversity during domestication process, we detected similar *π* values for the wild (1.85 × 10^−3^) and cultivated groups (1.62 × 10^−3^) in our germplasm diversity panel. We further computed the *F*_ST_ value between *Z*.* acidojujuba* and *Z*.* jujuba* accessions, which revealed that these groups showed low genetic differentiations (*F*_ST_ = 0.084) compared with citrus (*F*_ST_ = ~0.28) (Wang *et al*., 2017) and peach (*F*_ST_ = ~0.76) (Li *et al*., 2019). These observations of similar nucleotide diversity and low genetic differentiation between wild and cultivated groups can potentially be explained by relative weak artificial selective pressure during domestication, perhaps owing to jujube's relatively short period of intensive breeding efforts and great difficulty in artificial cross‐breeding (Li and Wei, 2013; Liu *et al*., 2016).

Our study provides rich data to support inferences about the evolutionary and domestication history of jujube. Combining population genomic analyses and archaeological evidence, our results support a previous hypothesis that jujube originated in the middle and lower reaches of the Yellow River—where the Shanxi–Shaanxi Gorge was likely the earliest cultivation centre (Qu and Wang, 1993; Liu, 2006)—and also support the new idea that Shanxi was likely the earlier centre of domestication as compared to Shaanxi. The genomic analyses revealed that *Z*.* acidojujuba* and *Z*.* jujuba* diverged around 2.7 Mya (Figure 3a), raising the suggestion that there may have been a long pre‐domestication period, perhaps similar to that reported for peach (Yu *et al*., 2018). Similarly, recent study of peach evolution also revealed that edible fruits of ancient peach had been enlarged compared with wild peach long prior to domestication based on peach endocarp fossils (2.6 Mya) found in southwest China (Yu *et al*., 2018).

The seed‐setting rate and fruit weight and are two important jujube domestication traits: the cultivated accessions have larger fruits with dramatically lower seed‐setting rates (Figures 1a and 4d and Figure S3). It is likely that humans intentionally selected jujubes with large fruit during domestication, but because of distinctive fruit anatomy and the fact that the number of seeds found inside a jujube's single kernel does not influence the eating quality, it is not probable humans selected jujubes with low seed‐setting rates. Specifically, humans were likely unaware of seed‐setting rates each because jujube fruit has only one hard kernel which contains two, one, or no seeds, which is distinct from the histories of other perennial fruit crops species such as wild banana (Sethiya *et al*., 2019) and wild citrus (Wang *et al*., 2017) for which many seeds in fruits make their consumption uncomfortable and inconvenient for people.

These reasons could help substantiate our idea that fruit size is a subjective target of selection, whereas seed‐setting rate is an accessary domestication trait. Perhaps some sexual reproduction disorder that induces embryogenesis failure in cultivars could result in more nutrients and photosynthate being partitioned to promote the development of the mesocarp, the desirable edible part of the fruit. Consistently, it has been shown that both fruit size and fruit drop are negatively impacted by inadequate nutrient supplies to each developing fruitlet in various perennial fruit crops including both citrus and Chinese jujube (Song *et al*., 2015; Sakhidin et al., 2019).

Our analyses identified candidate genes that have contributed to fruit weight and are likely responsible for seed‐setting during jujube domestication, and this information could help in fruit improvement efforts. Our genomic resequencing data substantially deepen the genetics resources available for both basic and applied research about this important fruit crop, and our analyses introduce multiple genetic hypotheses about how the metabolism and fruit quality traits have been altered during the domestication and subsequent dispersion and ongoing widespread cultivation of jujube.

## Experimental procedures

### Sample collection and agronomic evaluation

All jujube cultivars and two wild accessions were sampled in National Jujube Germplasm Repository of China, Pomology Institute, Shanxi Academy of Agricultural Sciences (Taigu county, Shanxi Province, China). The other 200 wild jujubes were gathered from wilderness such as low mountains, hills, river valleys, Gobi desert, edges of fields and road sides. The geographic distributions of the sampled 493 accessions were from 20 provinces/autonomous regions/municipalities of China covering nearly all jujube‐planting areas, and two cultivars were collected from Republic of Korea (Figure 1b and Table S1).

For GWAS analysis, fruit weight was investigated and evaluated in 2016, 2017 and 2018. Seed‐setting rate was investigated in 2018. The phenotypic data of these two traits were collected in National Jujube Germplasm Repository of China and wilderness. These traits were characterized based on previously published ‘*Descriptors and Data Standard for Jujube* (*Ziziphus jujuba* Mill.)’ (Li, 2006). Briefly as follows, fruit weight was determined as the average value of ten fruits using electronic balance. Seed‐setting rate equals number of kernels with full seeds/number of all detected kernels and was evaluated using over 30 fruits.

### Library preparation and genome sequencing

The genomic DNA was extracted with a total amount of 500 ng per sample and used as input material for the DNA sample preparations. Sequencing libraries were generated using TruSeq Nano DNA HT Sample Preparation Kit (Illumina USA) following the manufacturer’s recommendations, and index codes were added to attribute sequences to each sample. These libraries were sequenced on the Illumina HiSeq X Ten platform to obtain 350‐bp paired‐end reads. To obtain reliable reads, the raw reads (fastq format) were subjected to a series of quality control procedures to remove the low‐quality reads. Quality control involved the following steps: (i) removing reads with ≥ 10% unidentified nucleotides (N); (ii) removing reads with >20% bases having Phred quality <5; (iii) removing reads with >10 nt aligned to the adapter, allowing ≤10% mismatches; and (iv) removing putative PCR duplicates generated by PCR amplification during the library construction process (i.e. read 1 and 2 of two paired‐end reads that were completely identical). Finally, we obtained 3.11 trillion high‐quality paired‐end reads.

### Variation calling

The remaining high‐quality paired‐end reads were mapped to the *Ziziphus jujuba* reference genome using BWA (Burrows–Wheeler Aligner) (Version: 0.7.8) (Li *et al*., 2009) with the command ‘mem ‐t 4 ‐k 32 –M’. The alignment results were converted to BAM files using SAMtools software (Li *et al*., 2009). In order to reduce mismatch generated by PCR amplification before sequencing, duplicated reads were removed by SAMtools (Li *et al*., 2009). After alignment, we performed SNP calling on a population scale using a Bayesian approach as implemented in the package SAMtools (Li *et al*., 2009). We then calculated genotype likelihoods from reads for each individual at each genomic location, and the allele frequencies in the sample with a Bayesian approach. The ‘mpileup’ command was used to identify SNPs with the parameters as ‘‐q 1 ‐C 50 ‐S ‐D ‐m 2 ‐F 0.002 –u’. Then, to exclude SNP calling errors caused by incorrect mapping or indels, only high‐quality SNPs (coverage depth ≥ 6, RMS mapping quality ≥ 20, maf ≥ 0.05, miss ≤ 0.1) were kept for subsequent analysis. Indel calling was similar to SNP calling, and only those ≤ 5 bp were taken into account.

### Functional annotation of genetic variants

SNP/indel annotation was performed according to the *Ziziphus jujuba* genome using the package ANNOVAR (Wang *et al*., 2010). Based on the genome annotation, SNPs/indels were categorized as being located in exonic regions (overlapping with a coding exon), splicing sites (within 2 bp of a splicing junction), 5' UTRs and 3' UTRs, intronic regions (overlapping with an intron), upstream and downstream regions (within a 1‐kb region upstream or downstream from the transcription start site), and intergenic regions. The SNPs/indels in coding exons were further grouped into synonymous or nonsynonymous mutations. The SNPs causing gain of a stop codon, loss of a stop codon or splicing were designated as large‐effect SNPs. We further classified indels in coding exons as frameshift deletions or nonframeshift deletions.

### Phylogenetic tree and population structure

We selected a total of 1 952 561 high‐quality SNPs for phylogenetic and population structure analyses. To clarify the phylogenetic relationship from a genome‐wide perspective, an individual‐based neighbour‐joining (NJ) tree was constructed using the software TreeBest v1.9.2 (Vilella *et al*., 2009) with 1000 bootstraps and *Ziziphus mauritiana* was used as the outgroup. We further conducted principal component analysis (PCA) to evaluate genetic structure using the software GCTA (Yang *et al*., 2011), and the significance level of the eigenvector was determined using the Tracey–Widom test. Moreover, the population genetic structure was examined via an expectation maximization algorithm, as implemented in the program ADMIXTURE 1.23 (Alexander *et al*., 2009), and the number of assumed genetic clusters *K* ranged from 2 to 5 with 10 000 iterations for each run.

### Linkage disequilibrium analysis

To estimate and compare the pattern of linkage disequilibrium (LD) for wild, cultivated and total accessions, the squared correlation coefficient (*r* ^2^) between pairwise SNPs was computed using the software Haploview (Barrett *et al*., 2005). Parameters in the program were set as: ‘‐n ‐dprime ‐minMAF 0.05’. The average *r* ^2^ value was calculated for pairwise markers in a 500‐kb window and averaged across the whole genome, and LD decay figures for groups of genotypes were drawn using an R script.

### Divergence time

We selected one accession as representative from *Z*.* acidojujuba* (S206), *Z*.* jujuba* (Z12) and *Z*.* mauritiana* (Misi). *Prunus persica* (International Peach Genome Initiative *et al*., 2013), *Malus domestica* (Daccord *et al*., 2017) and *Pyrus bretschneideri* (Wu *et al*., 2013) were used to estimate the divergence time of *Z*.* acidojujuba*, *Z*.* jujuba* and *Z*.* mauritiana*. Raw data were downloaded from NCBI with accession number (PRJNA34817, PRJNA379390 and PRJNA157875), and the raw reads (fastq format) were subjected to a series of quality control procedures to remove the low‐quality reads, the method of quality control was described earlier (Library preparation and genome sequencing). The remaining high‐quality paired‐end reads were mapped to the *Ziziphus jujuba* reference genome using BWA (Burrows–Wheeler Aligner) (Version: 0.7.8) (Li *et al*., 2009), and SNP calling was performed using SAMtools (Li *et al*., 2009), the detailed method was described earlier (Variation calling). All fourfold degenerate sites were used to construct a phylogenetic tree, because these sites were considered as neutral evolution. Based on this phylogenetic tree, the MCMCtree program implemented in PAML was applied to infer the divergence time (Yang, 2007). The MCMCtree running parameters were as follows: model: JC69, burn‐in: 10 000, nsample: 100 000, sampfreq: 2. The calibration times of divergence between *Malus domestica* and *Pyrus bretschneideri*, *Malus domestica* and *Prunus persica*, and *Pyrus bretschneideri* and *Ziziphus jujuba* were 8–9, 40–43, and 86–88 Mya, respectively (Liu *et al*., 2014).

### PSMC analysis

The PSMC estimations were conducted using the default parameters tuned for human populations (Li and Durbin, 2011). S217, S357, S377, S442 and S447 for wild and Z12, Z78, Z95, Z232 and Z295 for cultivated groups were used for analysis. The utility fq2psmcfa provided with the PSMC software was used to convert this diploid consensus sequence to the required input format. The parameters were set as follows: ‘‐N30 ‐t15 ‐r5’. The generation time (*g*) was set as an estimate of two years based on our many years of field experiments, and a mutation rate (*μ*) was set as 1.38 × 10^−8^ substitutions per site per year (Xie *et al*., 2016).

### Selective sweep analysis

To identify genome‐wide selective sweeps, we scanned the genome in 50 kb sliding scales with a step size of 5 kb and calculated the genome‐wide distribution of fixation index (*F*_ST_) and the genetic diversity (*π*) in wild and cultivated breeds by vcftools (Danecek *et al*., 2011) implementing the Weir and Cockerham method, respectively. Then, we calculated the diversity ratio (*π*_wild_/π_cultivated_). The regions with *π*_wild_/π_cultivated_ values and *F*_ST_ scores in the top 5% were considered to have been subjected to selective sweeps, respectively.

In addition, we identified the genomic regions with allele frequency differentiation using the likelihood method (XP‐CLR) (Chen *et al*., 2010). We used a 0.05‐cM sliding window with 100‐bp steps across the whole genome. The maximum number of SNPs assayed in each window was 200, and the command line was XP‐CLR − c freqInput outputFile − w1 gWin(Morgan) snpWin gridSize(bp) chrN. Finally, we calculated the mean likelihood score in 50‐kb sliding windows with a step size of 5 kb across the genome. Windows with the top 5% of XP‐CLR values were selected and merged into regions.

### Genome‐wide association study (GWAS)

We carried out a large‐scale GWAS using the genotypic data from 1 952 561 common SNPs (minor allele frequency ≥ 0.05; missing rate ≤ 0.1, depth ≥ 6). Association analysis was conducted using the GEMMA (Zhou and Stephens, 2012) (genome‐wide efficient mixed‐model association) software package. For the MLM analysis, we used the equation

y=Xα+Sβ+Kμ+e.

In these equations, *y* represents phenotype, *X* represents genotype, *S* is the structure matrix, and *K* is the relative kinship matrix. *X*α and *S*β represent fixed effects, and *K*μ and *e* represent random effects. The top three PCs were used to build up the *S* matrix for population structure correction. The matrix of simple matching coefficients was used to build up the *K* matrix. The analyses were performed using GEMMA software package (Zhou and Stephens, 2012).

### Transcriptome analysis and qPCR

Raw data were downloaded from NCBI with accession number (for ‘Junzao’: SRX1518646, SRX1518647, SRX1518648, SRX1518650, SRX1518651, SRX1518652, SRX1518653, SRX1518654, SRX1518655 and SRX1518656; for ‘Dongzao’: SRX691543, SRX691544, SRX691546 and SRX691547; and for one wild accession: SRX1518707, SRX1518708, SRX1518709 and SRX1518710); then, clean data were obtained by removing reads containing adapter, reads containing ploy‐N and low‐quality reads from raw data, and these high‐quality reads were mapped to the draft reference genomes by HISAT with the parameters: ‐‐max‐intron‐length 500000 (Kim *et al*., 2015). The expression level (FPKM value) for each protein‐coding gene was calculated by cufflinks (https://github.com/cole‐trapnell‐lab/cufflinks).

For qPCR, total RNA was extracted using Takara MiniBEST Plant RNA Extraction l. The first‐strand cDNA was synthesized according to the manufacturer’s protocol (Promega, Madison). qPCR was performed in triplicate with the SYBR Green I Master (DBI) using *ZjActin* (GenBank: KT381859) and *SlUbiquitin* (*Solyc07g064130*) as the endogenous. The relative quantification method (2^‐ΔΔCT^) was used to evaluate quantitative variation between replicates examined (Livak and Schmittgen, 2001). Primers used for qPCR are listed in Table S14.

### Plasmid construction and transgenic experiments

The full‐length coding sequence of *ZjDA3^Hap^* ^.^ *^A^* (*Zj*.*jz038707057*) gene was amplified using jujube fruit cDNA. The amplified products were further cloned into the modified pCAMBIA‐1302 vector driven by the cauliflower mosaic virus (CaMV) 35 S promoter. *Solanum lycopersicum* cv. Micro‐Tom tomato was used as the receptor plant for transformation by *Agrobacterium tumefaciens* according to the previous study (Fillatti *et al*., 1987). The T0 transgenic plants were generated and grown to maturity in pots under glasshouse conditions to evaluate fruit size and measure gene expression. Primers used for vector construction are listed in Table S14.

### Data access

All the genomic sequence data sets for genetic diversity analysis and GWAS have been deposited in the NCBI Sequence Read Archive under accession numbers PRJNA560664 and PRJNA563262.

## Conflict of interest

The authors declare there is no conflict of interest.

## Author contributions

M.G., X.Z., Z.Z., S.L., Q.L., P.F., W.J., J.Z. and R.L. conceived and designed the project. M.G., S.L. and X.Z. collected samples for DNA sequencing. M.G., S.L., Y.H., J.Q., K.X., L.L., M.W., Z.D., S.L., J.W., P.S., Q.Y., G.X., D.L., Y.W. and S.T. performed field experiments and phenotyping. Z.Z., M.G., J.Z., W.J. and X.F. performed sequencing, genomic variant, population analysis and GWAS analyses. M.G., and Z.Z. performed data integration and results analyses. M.G. conducted gene expression analysis and transgenic experiments. M.G. and Z.Z. wrote the manuscript. All authors read and approved the manuscript.

## Supporting information


**Table S1** Summary of 493 jujube accessions in this study.

**Table S2** Genome resequencing data of 493 jujube accessions.

**Table S3** Whole‐genome SNP and Indel information.

**Table S4** Sanger sequencing on genomic regions of randomly selected SNPs.

**Table S5** List of candidate domestication regions by π ratio, *F*_ST_ and XP‐CLR.

**Table S6** Genome‐wide screening of genes within selective sweep regions via π ratio, *F*_ST_ and XP‐CLR analyses.

**Table S7** Gene ontology (GO) categories assigned to the genes identified through overlapping π ratio and *F*_ST_ analyses.

**Table S8** List of genes in the domestication region highly associated with reproduction system and fruit size.

**Table S9** List of candidate genes associated with fruit weight harbored in two GWAS peaks on chromosome 1.

**Table S10** List of the representative accessions of fresh and dry jujubes used in *F*_ST_ and XP‐CLR analyses.

**Table S11** List of candidate selection regions by *F*_ST_ and XP‐CLR between fresh and dry jujubes.

**Table S12** Genome‐wide screening of genes within selective sweep between fresh and dry jujubes.

**Table S13** List of genes in the selection region associated with fruit sweetness and crispness.

**Table S14** Primers used in this study were listed.

**Figure S1** The phenotypes of wild and cultivated jujubes.
**Figure S2** Gene ontology (GO) categories assigned to the 993 candidate genes identified through overlapping two approaches, *π*_w/_ *π*_c_ and *F*_ST_.
**Figure S3** Box plots for seed‐setting rate (left) and fruit weight (right) of wild and cultivated jujubes.
**Figure S4** GWAS for seed‐setting rate.
**Figure S5** PCR amplification and Sanger sequencing of *ZjPOD1* and *ZjDA3* were conducted to validate the variations.
**Figure S6** GWAS for fruit weight using different populations.
**Figure S7** Statistical data of tomato fruit length and width in wild type (WT) and three overexpression of *ZjDA3* lines.
