# Supplementary material for: Genomic analyses of diverse wild and cultivated accessions provide insights into the evolutionary history of jujube
Source: Plant Biotechnol J. 2020 Sep 30;19(3):517–31. doi: 10.1111/pbi.13480 (PMC7955879; doi:10.1111/pbi.13480)
Supplement: Supplementary file 15 — Figure S1 The phenotypes of wild and cultivated jujubes. Figure S2 Gene ontology (GO) categories assigned to the 993 candidate genes identified through overlapping two approaches, π w/ π c and F ST. Figure S3 Box plots for seed‐setting rate (left) and fruit weight (right) of wild and cultivated jujubes. Figure S4 GWAS for seed‐setting rate. Figure S5 PCR amplification and Sanger sequencing of ZjPOD1 and ZjDA3 were conducted to validate the variations. Figure S6 GWAS for fruit weight using different populations. Figure S7 Statistical data of tomato fruit length and width in wild type (WT) and three overexpression of ZjDA3 lines. [file PBI-19-517-s014.docx]

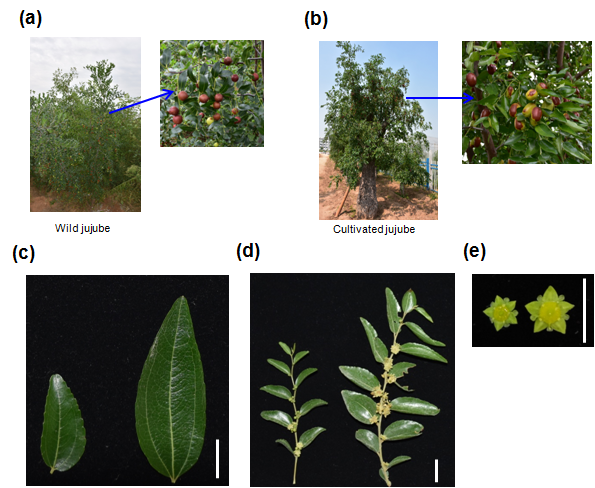


**Figure S1** The phenotypes of wild and cultivated jujubes. (a and b) The intact plants and fruits of wild jujube and cultivated jujube. (c-e) Leaves, bearing shoots and flowers of wild and cultivated jujubes. Left, wild; Right, cultivated. (c and e) Bars = 1 cm. (d) Bar = 2 cm.


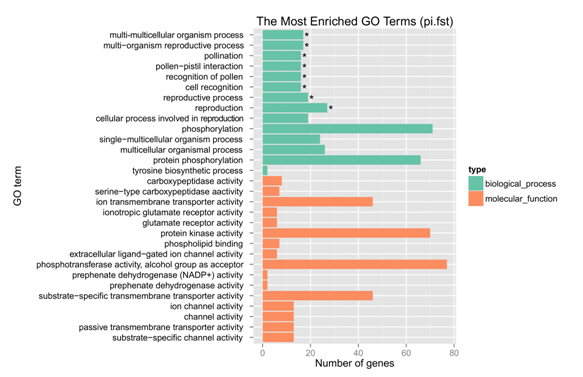


**Figure S2** Gene ontology (GO) categories assigned to the 993 candidate genes identified through overlapping two approaches, *π*_w/_*π*_c_ and *F*_ST_. The *x* axis represents the number of genes, and the *y* axis indicates the GO subcategories.


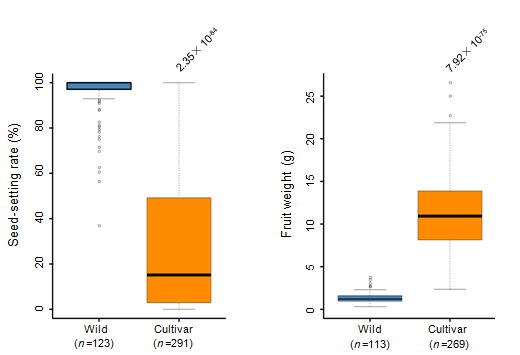


**Figure S3** Box plots for seed setting rate (left) and fruit weight (right) of wild and cultivated jujubes. Center bold lines indicate the median, and box limits represent the upper and lower quartiles. Whiskers extend to data no more than 1.5 times the interquartile range, and dots represent outliers. *n* indicates the number of accessions with the same type. Significant differences were determined by two-tailed Welch’s *t* test.


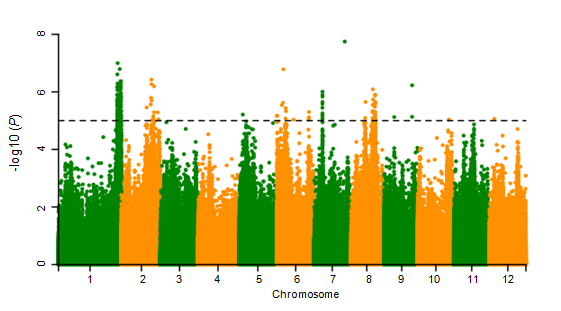


**Figure S4** GWAS for seed-setting rate. Dashed lines represent significance thresholds (−log_10_ *P* = 5).


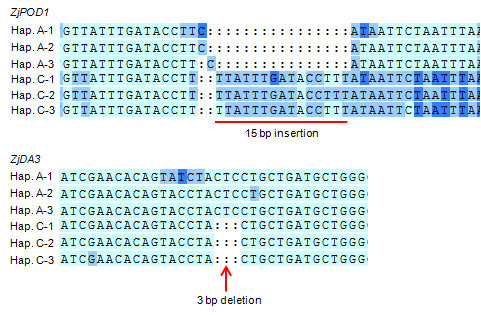


**Figure S5** PCR amplification and Sanger sequencing of *ZjPOD1* and *ZjDA3* were conducted to validate the variations. Three accessions carrying haplotype A and C were selected, respectively. (Top) *ZjPOD1* results. (Bottom) *ZjDA3* results.


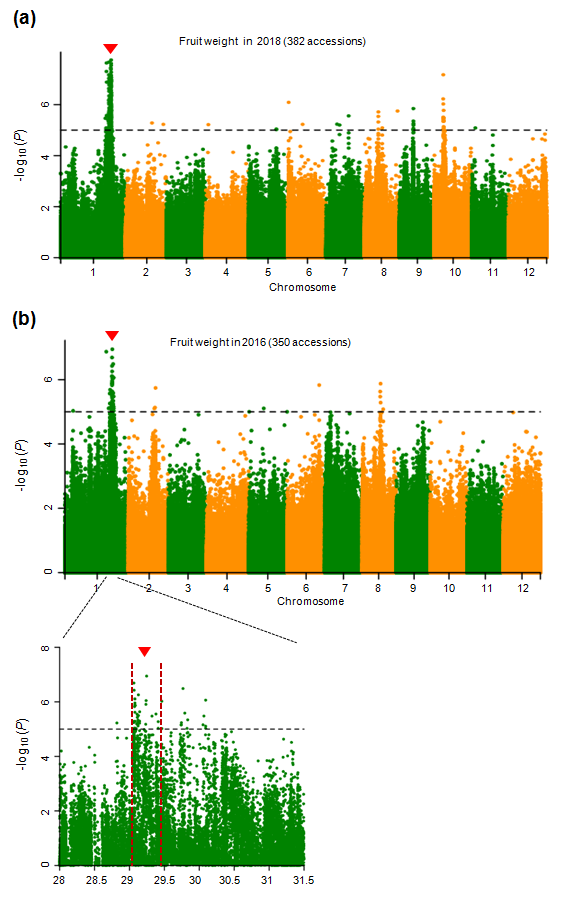


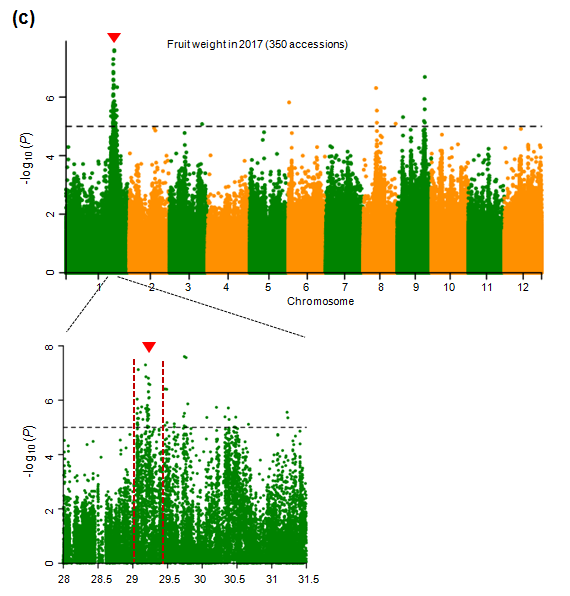


**Figure S6** GWAS for fruit weight using different populations. (a) Manhattan plots for fruit weight using 382 accessions. (b) Manhattan plots for fruit weight using 350 accessions in 2016 year. (c) Manhattan plots for fruit weight using 350 accessions in 2017 year. Red arrowheads indicate the position of peaks identified in this study. Dashed lines represent significance thresholds (−log_10_ *P* = 5).


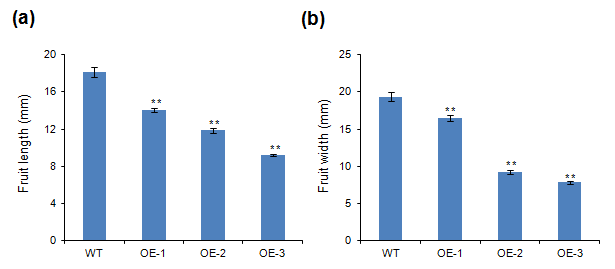


**Figure S7** Statistical data of tomato fruit length and width in wild type (WT) and three overexpression of *ZjDA3* lines. (a) Fruit length. (b) Fruit width. Values are means± SE, *n* = 8. ‘**’ indicated *P* < 0.01.
